# The Effectiveness of the Smartphone-Based WeChat Platform on Reducing Time to Diagnosis and Treatment of ST-segment Elevation Myocardial Infarction

**DOI:** 10.31083/j.rcm2412374

**Published:** 2023-12-29

**Authors:** GuanYang Kang, HuiQing Zhang, Jian Zhou, DeLi Wan

**Affiliations:** ^1^Department of Cardiology, Bin Hai Wan Central Hospital of Dongguan (also called The Fifth People's Hospital of Dongguan, Taiping People's Hospital of Dongguan), 523905 Dongguan, Guangdong, China; ^2^Department of Clinical Pharmacy, Bin Hai Wan Central Hospital of Dongguan (also called The Fifth People's Hospital of Dongguan, Taiping People's Hospital of Dongguan), 523905 Dongguan, Guangdong, China; ^3^Department of Cardiovascular Medicine, Shanghai East Hospital, Tongji University School of Medicine, 200123 Shanghai, China

**Keywords:** myocardial infarction, telemedicine, WeChat platform, mobile health, social media

## Abstract

**Background:**

This study evaluated the effectiveness of the smartphone-based WeChat platform in reducing the ischemia time of ST-segment elevation myocardial infarction (STEMI).

**Methods:**

A total of 198 STEMI patients who underwent primary percutaneous coronary intervention (PCI) from January 2022 to August 2022 in our hospital were enrolled in this retrospective cohort study. Patients were divided into two groups according to whether their electrocardiograms (ECGs) were posted on the WeChat platform. The two groups were compared for the following: diagnosis time of first ECG, time from first medical contact (FMC) to catheterization laboratory (CL) activity, bypass emergency department (ED) or critical care unit (CCU), time of door to wire, time of door to balloon, time of FMC to wire, heart failure during hospitalization, cardiogenic shock during hospitalization, malignant arrhythmia during hospitalization, death during hospitalization, total hospital cost, and length of stay.

**Results:**

The diagnosis time for the first ECG was 10.05 ± 3.30 mins in the control group and 2.50 ± 0.82 mins in the WeChat group (*p* < 0.05). The time from FMC to CL activity was significantly shorter in the WeChat group compared to the control group (*p* < 0.05). None of the control group patients bypassed the ED, compared to 80 (80%) of patients in the WeChat group (*p* < 0.05). The time from door to wire was 60.22 ± 12.73 mins in the WeChat group and 92.56 ± 20.23 mins in the control group (*p* < 0.05). The time of FMC to wire was also significantly shorter in the WeChat group than in the control group (*p* < 0.05). The WeChat group had a significantly lower rate of heart failure during hospitalization than the control group (*p* < 0.05). However, the two groups showed no significant differences for cardiogenic shock during hospitalization, malignant arrhythmia during hospitalization, death during hospitalization, total hospital cost, and length of stay.

**Conclusions:**

The smartphone-based WeChat platform demonstrated high efficacy and accessibility in reducing the ischemia time for STEMI patients. Our results indicate that social media platforms such as WeChat could be a useful approach for improving the prognosis of cardiovascular disease.

## 1. Introduction

ST-segment elevation myocardial infarction (STEMI) is a medical emergency whereby the survival of patients and their clinical outcome relies on minimizing total ischemic time between the onset of symptoms and reperfusion [1]. A major predictor of death in STEMI patients who undergo primary percutaneous coronary intervention (PCI) is system delay [2]. Guidelines from the American College of Cardiology Foundation/American Heart Association and the European Society of Cardiology stress the significance of reducing the period between first medical contact (FMC) and PCI to 90 mins or less [3, 4, 5, 6]. Over the past few decades, the implementation of a number of essential strategies that target system delays has resulted in significantly improved management of STEMI patients. These strategies include the acquisition of a pre-hospital electrocardiogram (ECG) by emergency medical services, and activation of the cardiac catheterization laboratory (CL) [7, 8, 9]. The latter is essential for lowering the reperfusion time and is related to reduced mortality, but may lead to numerous cancellations due to false activation [9]. The past few years have seen the successful introduction of telemedicine systems, social media platforms, and mobile applications to improve the outcomes for cardiovascular disease patients [10, 11, 12].

WeChat is a Chinese instant messaging and social networking platform developed by Tencent, a Chinese technology firm. WeChat had more than a billion monthly active users in 2018, which made it the largest standalone mobile application in the world. It has been called China’s “app for everything” and a “super-app” because of its extensive functionality. WeChat offers messaging by voice, text and broadcast, teleconferencing, and the sharing of videos and photos. WeChat also allows users to easily participate in group conversations, add new members, and perform various other functions without the need to call or email. A number of studies have reported on WeChat’s efficacy for the management of chronic disease [10, 11].

WeChat may offer a unique opportunity to solve the present shortage of telemedicine services offered to Chinese STEMI patients thanks to its many functionalities and very large user base. However, only limited research has so far been carried out to evaluate the smartphone-based WeChat platform as a strategy for reducing the ischemia time of STEMI. In 2019, our hospital, together with five hospitals lacking PCI capacity, launched a chest pain center. The WeChat application was downloaded and installed on a smartphone. A WeChat platform titled “Dongguan Chest Pain Center” was then launched. The aim of this work was to evaluate the smartphone-based WeChat platform for its effectiveness in reducing the ischemia time of STEMI.

## 2. Methods

### 2.1 Participants

This retrospective cohort study enrolled 198 acute STEMI patients who had undergone primary PCI at Bin Hai Wan Central Hospital, Dongguan, from January 2022 to August 2022. Our hospital features a PCI center that provides PCI reperfusion therapy for 24 hours per day and every day of the week. In 2019, our hospital, together with five hospitals that lacked PCI capacity, launched a chest pain center. These 5 non-PCI hospitals are located 3 to 35 kilometers away from our PCI center, and the average transfer time to our PCI center is between 10 and 35 mins.

**Fig. 1.**
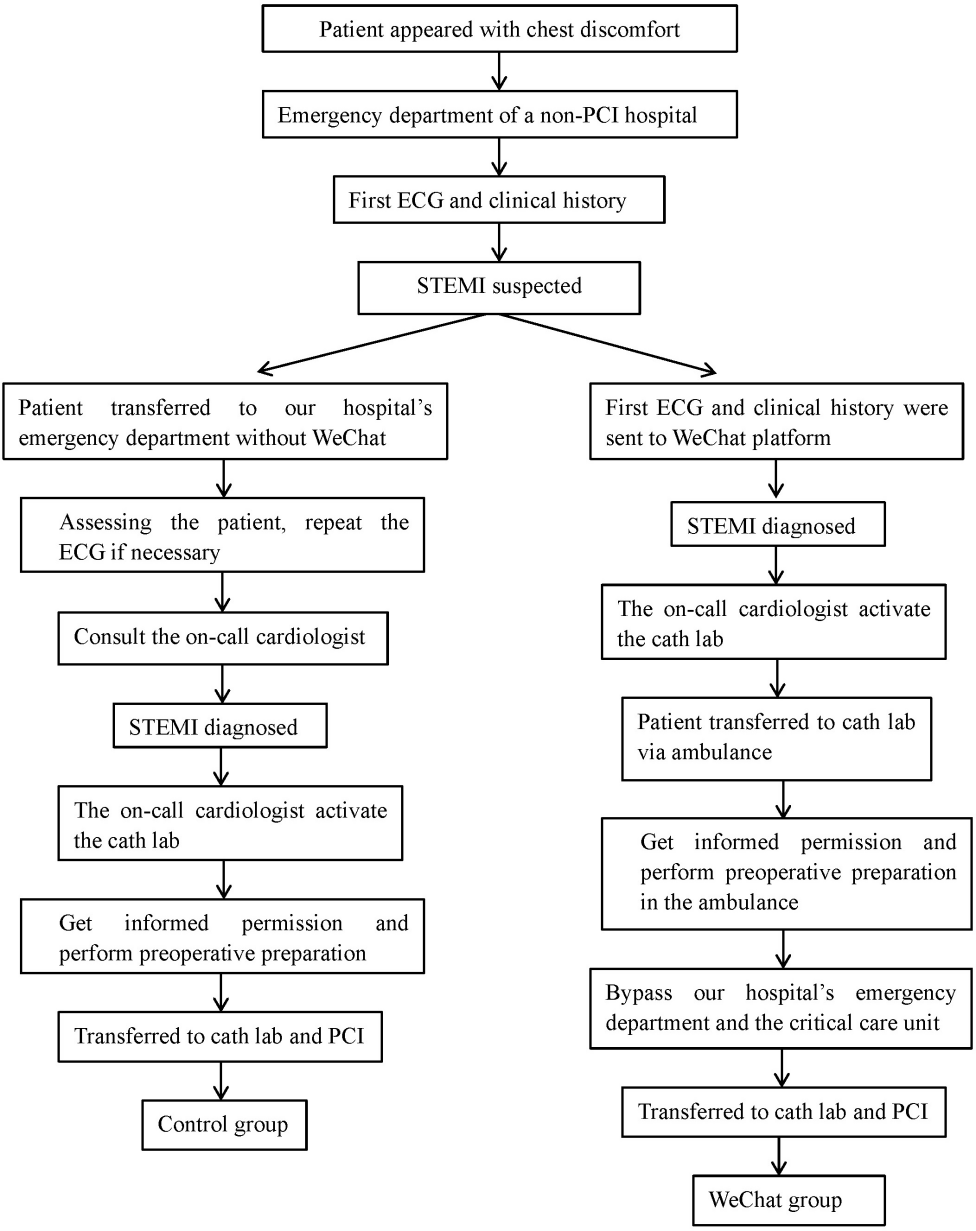
**Study design**. PCI, percutaneous coronary intervention; ECG, electrocardiogram; STEMI, ST-segment elevation myocardial infarction.

Patients were classified into two groups according to whether their ECGs were posted on the WeChat platform (Fig. 1).

#### 2.1.1 Control Group (n = 98) 

Patients in this group were treated according to the standard emergency green channel. In China, this is a rapid, efficient and standardized service provided for critically ill patients in hospitals without PCI technology. When patients present with chest pain in the emergency department (ED) of non-PCI hospitals, the FMC physician performs and analyzes the initial ECG. If STEMI was suspected in the ED at one of the non-PCI institutions, the patient was immediately transferred to our hospital. Here, the emergency room physician performed a repeat ECG if necessary, made a preliminary diagnosis, and consulted with the on-call cardiologist. The cardiologist then examined the patient’s clinical history and ECG during their visit to the ED. If STEMI was diagnosed, the cardiologist requested a consent form for PCI from the patient. They also contacted nurses to activate the CL and informed PCI clinicians by phone to prepare for the procedure. Patients were then transferred to the CL for PCI. Patients in the control group did not have their ECGs uploaded to WeChat.

#### 2.1.2 WeChat Group (n = 100)

**Fig. 2.**
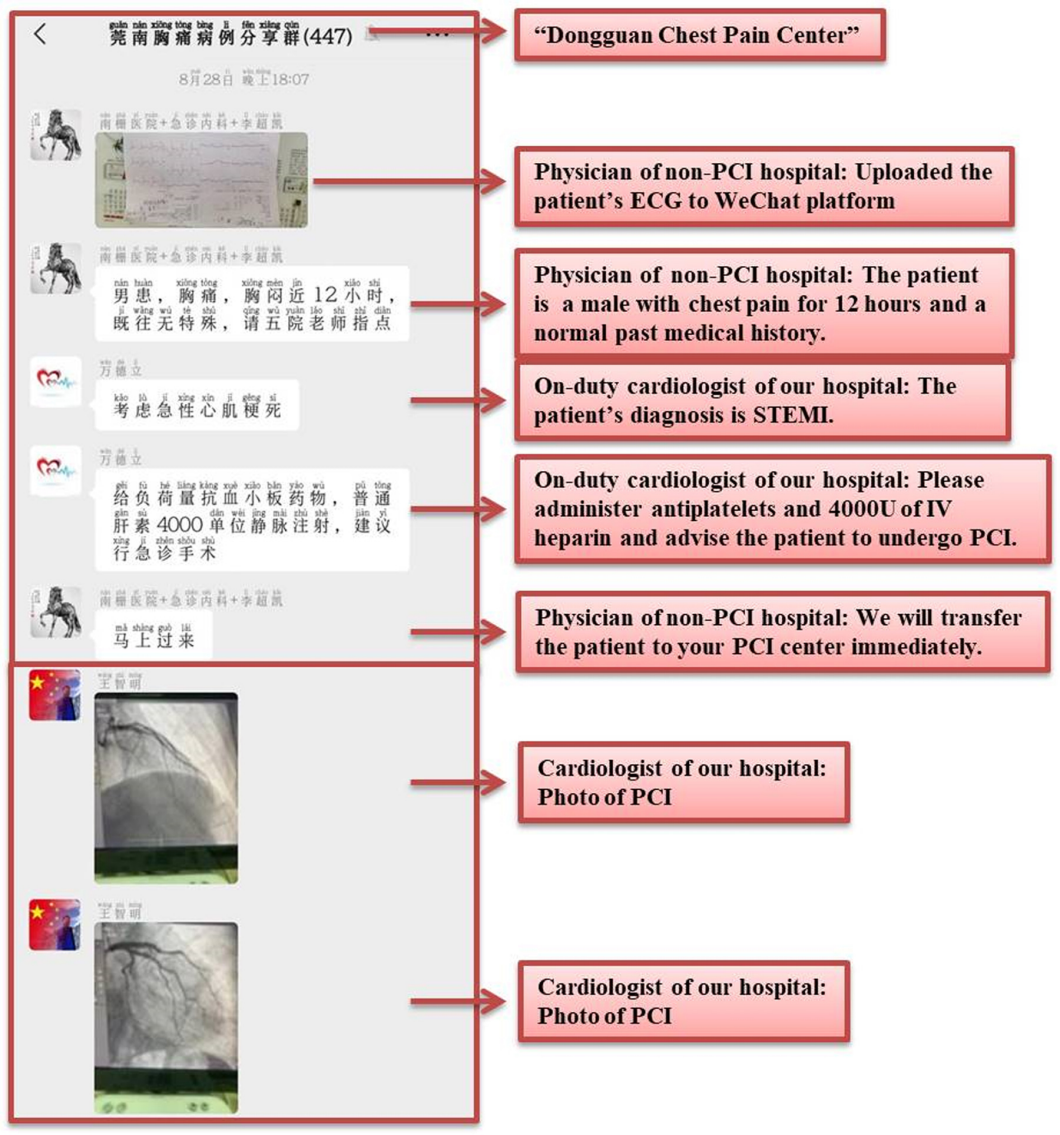
**Screenshots of the smartphone-based WeChat platform**. IV, intravenous; ECG, electrocardiogram; PCI, percutaneous coronary intervention; STEMI, ST-segment elevation myocardial infarction.

In 2019, our hospital and five non-PCI hospitals established a chest pain center. The WeChat application was downloaded, installed on smartphones and a WeChat platform titled “Dongguan Chest Pain Center” was launched. An invitation to join this platform was extended to 447 physicians, nurses, and other healthcare professionals from our hospital and the non-PCI hospitals. In the WeChat group, when a patient presented with chest discomfort in the ED of non-PCI hospitals, the FMC physician performed and evaluated an initial ECG. If this showed ST changes, the ECG results were promptly uploaded onto the WeChat platform using a smartphone and were analyzed by the on-duty cardiologist at our PCI center. Their evaluation was posted on the WeChat platform within ten mins. If STEMI was diagnosed, the non-PCI hospital rapidly transferred the patient to our PCI center via ambulance. While in the ambulance, the FMC physician and nurse obtained informed consent from the patient and carried out preoperative preparations. Meanwhile, the on-call cardiologist at our hospital triggered the CL and informed PCI physicians by WeChat and phone that they were ready. When the patient arrived at our PCI center, they bypassed the ED and critical care unit (CCU) and went directly to the CL for interventional treatment. The on-call cardiologist at our hospital was sometimes unable to establish a definitive diagnosis from the initial ECG sent by the FMC physician from the non-PCI hospital via WeChat. In this situation, the patient was sent to our hospital’s ED for further evaluation. If the patient arrived at our hospital’s ED with chest pain, they would bypass the CCU and proceed directly to the CL for interventional treatment. No patient identifiers were revealed on WeChat. Within two hours of revascularization, all information sent via WeChat was deleted by the platform manager using the application program’s remote data-wiping function. The FMC physician received informed consent from the patient before disclosing their ECG results. In addition, patient privacy and data protection rules apply in our hospital. Fig. 2 shows various screenshots of the WeChat platform that outline this process. Patient ECGs were uploaded to the platform in the WeChat group.

Baseline clinical characteristics were collected from each patient using a questionnaire and were obtained from medical records. These included the patient demographic, medical history, medical insurance, smoking history, education level, household income, marital status, Killip classification, intra-aortic balloon pump therapy, and time of onset to FMC. The relevant healthcare providers recorded critical time points using a dedicated “time management form for patients with chest pain”. These times included the onset of symptoms, FMC, initial ECG, ECG diagnosis, arrival at our hospital, CL activation, wire crossing, and door to balloon. All time-point definitions followed 2017 European Society of Cardiology (ESC) guidelines for the management of acute myocardial infarction in patients with ST-segment elevation. Control and WeChat groups were compared for the following parameters: diagnosis time after the first ECG (time taken to establish a definitive diagnosis following the initial ECG), FMC to CL activity, bypass ED or CCU, door to wire, FMC to wire, heart failure during hospitalization, cardiogenic shock during hospitalization, malignant arrhythmia during hospitalization, death during hospitalization, total hospital cost, and length of stay.

### 2.2 Statistical Analyses

SPSS version 20.0 (IBM Corp., Armonk, NY, USA) was employed for analysis of the data. Basic patient characteristics were presented as the frequency, and mean ± standard deviation (SD). Baseline clinical characteristics for control and WeChat groups were compared with χ^2^ test for categorical data and independent *t*-test for continuous variables. Non-parametric data was evaluated using Wilcoxon signed-rank sum test. *p* values < 0.05 were considered to indicate a statistically significant difference.

## 3. Results

As shown in Table 1, none of the baseline characteristics were significantly different between control and WeChat groups (*p* > 0.05).

**Table 1.** **Baseline demographic and clinical characteristics of participants**.

Variable		Control (n = 98)	WeChat (n = 100)	*p*-value
Mean age (years)		56.7 ± 12.2	57.0 ± 11.9	0.86
Male		80 (81.63)	82 (82.00)	0.95
Female		18 (18.37)	18 (18.00)	0.95
BMI (kg/$m^{2}$)		26.1 ± 3.3	26.0 ± 3.2	0.83
Race				
	Han	95 (96.94)	97 (97.00)	0.70
	Minority	3 (3.06)	3 (3.00)	0.70
Smoking history				
	Yes	65 (66.33)	67 (67.00)	0.92
	No	33 (33.67)	33 (33.00)	0.92
Schooling (years)		12.0 ± 3.4	11.7 ± 3.5	0.54
Monthly household income				
	<¥3000 ($410)	30 (30.61)	31(31.00)	0.95
	¥3000–¥5000 ($410–$683)	50 (51.02)	52(52.00)	0.89
	>¥5000 ($683)	18 (18.37)	17(17.00)	0.80
Marital status				
	Married	90 (91.84)	90 (90.00)	0.65
	Other	8 (8.16)	10 (10.00)	0.65
Killip classification				
	I	70 (71.43)	69 (69.00)	0.71
	II	10 (10.20)	10 (10.00)	0.96
	III	10 (10.20)	12 (10.00)	0.69
	IV	8 (8.16)	9 (9.00)	0.83
Medical insurance				
	Yes	90 (91.84)	90 (90.00)	0.65
	No	8 (8.16)	10 (10.00)	0.65
Medical history				
	Hyperlipidemia	50 (51.02)	51 (51.00)	1.00
	Hypertension	56 (57.14)	55 (55.00)	0.76
	Diabetes mellitus	26 (26.53)	27 (27.00)	0.94
	Prior coronary disease	15 (15.31)	15 (15.00)	0.95
	IABP therapy	2 (2.04)	2 (2.00)	0.63
Onset to FMC				
	≤2 hours	50 (51.02)	50 (50.00)	0.89
	>2 hours	48 (48.98)	50 (50.00)	0.89

Values shown are the number (%), or mean ± standard deviation (SD). IABP, intra-aortic balloon pump; FMC, first medical contact; BMI, body mass index.

As shown in Table 2, the diagnosis time after the first ECG was 10.05 ± 3.30 mins in the control group and 2.50 ± 0.82 mins in the WeChat group (*p* < 0.05). The time of FMC to CL activity in the WeChat group was shorter than in the control group (*p* < 0.05). None of the control group patients bypassed the ED, compared to 80 (80%) patients in the WeChat group (*p* < 0.05). Time from door to wire was 92.56 ± 20.23 mins in the control group and 60.22 ± 12.73 mins in the WeChat group (*p* < 0.05). The WeChat group had a significantly shorter FMC to wire time than the control group (*p* < 0.05), and reduced heart failure rate during hospitalization (*p* < 0.05). However, the two groups showed no significant differences for cardiogenic shock during hospitalization, malignant arrhythmia during hospitalization, death during hospitalization, total hospital cost, and the length of hospital stay (*p* > 0.05).

**Table 2.** **Comparison of outcomes between control and WeChat groups**.

Outcome		Control (n = 98)	WeChat (n = 100)	*p*-value
Diagnosis time of first ECG (min)		10.05 ± 3.30	2.50 ± 0.82	<0.01
FMC to CL activity (min)		30.18 ± 9.25	12.80 ± 5.12	<0.01
Bypass ED or CCU		0 (0)	80 (80)	<0.01
Door to wire (min)		92.56 ± 20.23	60.22 ± 12.73	<0.01
Door to balloon (min)		98.21 ± 21.02	65.92 ± 13.86	<0.01
FMC to wire (min)		128.28 ± 20.22	98.56 ± 18.26	<0.01
Event during hospitalization				
	Heart failure	15 (15.31)	6 (6.00)	0.03
	Cardiogenic shock	5 (5.10)	6 (6.00)	0.78
	Malignant arrhythmia	10 (10.20)	9 (9.00)	0.77
	Death	5 (5.10)	4 (4.00)	0.97
Total hospital cost (¥)		31,807 ± 600 ($4345 ± $82)	31,752 ± 608 ($4338 ± $83)	0.52
Length of stay (days)		7.21 ± 1.29	7.02 ± 1.32	0.31

The values shown are the number (%), or mean ± standard deviation (SD). ECG, electrocardiogram; FMC, first medical contact; ED, emergency department; CCU, critical care unit; CL, catheterization laboratory.

## 4. Discussion

The goal of this retrospective cohort analysis was to assess how effective the smartphone-based WeChat platform was in reducing the ischemia time of STEMI patients. Our results showed the smartphone-based WeChat platform can significantly reduce the times for first ECG diagnosis, FMC to CL activity, door to wire, and FMC to wire. Furthermore, the application of the smartphone-based WeChat platform led to a significantly lower rate of heart failure during hospitalization compared to the control group. These results support the use of social media as an effective strategy for reducing the time to diagnosis and treatment, improving the prognosis of STEMI patients.

Due to technological advances and the ubiquity of smartphones, WeChat has become the most popular social networking and messaging app in China. It is economical, rapid, and allows face-to-face communication. WeChat has become the primary mode whereby individuals can obtain and exchange information. Medical and healthcare services are also currently accessible through this platform. It has already been reported that WeChat is highly effective for chronic disease management following hospital discharge [10, 11, 12].

Despite years of improvement, the timely diagnosis of STEMI patients in the ED poses considerable practical challenges and still relies mainly on ECG. Obtaining and diagnosing the ECG within less than 10 mins of arrival at ED are vital for achieving optimal outcomes in STEMI patients [13, 14, 15]. When patients in the WeChat group of our study complained of chest pain and were sent to ED for treatment, the FMC doctor obtained the initial ECG and carried out the primary analysis. If ST changes were found in this initial ECG, the images were promptly sent via the WeChat platform by smartphone and analyzed by an on-duty cardiologist at our PCI center. Their report was in turn promptly returned to the WeChat platform within 10 mins. Our study found that the use of the smartphone-based WeChat platform could significantly reduce the time of first ECG diagnosis compared to the control group. Moreover, our findings indicated that ECG transfer via the WeChat platform results in earlier reperfusion of STEMI patients.

In pre-hospital scenarios, rapid CL activation as soon as a STEMI diagnosis was made reduced treatment delays and possibly also mortality. Our study found that the smartphone-based WeChat platform greatly decreased the time between the FMC and CL activity. Using this platform, FMC doctors can rapidly post the chest pain history of patients and their ECG photos. This allows the on-call cardiologist in our PCI hospital to rapidly provide consultation, determine if interventional treatment is required, and trigger the CL. All of the essential personnel such as interventional physicians and nurses wait for the patient to arrive based on the information provided in the WeChat platform. The time from FMC to CL activity in the WeChat group was therefore significantly shorter than the control group. These results support those of Liu *et al*. [12] who reported that FMC to CL activity was shorter in the WeChat group than in the control group (29 mins and 74 mins, respectively; *p* < 0.001).

Numerous earlier studies reported that bypassing the ED and transporting patients directly to the CL can reduce reperfusion periods for STEMI patients prior to hospital admission. It has been reported that bypassing the ED can result in a saving of 20 mins between FMC and wire-crossing [16, 17]. The present study also showed the smartphone-based WeChat platform can significantly reduce the times for door to wire, and FMC to wire. Furthermore, STEMI patients in the WeChat group showed a significantly lower heart failure rate during hospitalization than patients in the control group. These research findings are in line with those of earlier studies [12, 18, 19, 20]. However, bypassing ED is only possible if the pre-hospital ECG was received and diagnosis was made prior to the patient’s arrival at the PCI hospital. The current study shows that a smartphone-based WeChat platform can facilitate information exchange and pre-hospital diagnosis by facilitating rapid transmission of ECG results. This allows STEMI patients to be sent directly to CL, thereby bypassing ED and CCU. Our findings support the conclusion that telemedicine interventions can have positive effects on the outcome of cardiovascular disease. Even with the prompt PCI, some patients had a discouraging prognosis. According to a number of studies, inflammation is both a cause and an aggravating factor in cardiovascular disease, as well as a mediator of its worst prognostic [21, 22, 23]. New inflammatory biomarkers should be analyzed to evaluate therapeutic efficacy in patients with STEMI in future studies.

The present study did not find any significant differences between WeChat and control groups for cardiogenic shock during hospitalization, malignant arrhythmia during hospitalization, or death during hospitalization. The reasons for this may include sample differences and the relatively short observation time. The follow-up period was also short and this may have affected the accuracy of our conclusions. Future studies should carry out long-term observations on the efficacy of chest pain centers for ameliorating the outcome of STEMI patients. The current study was performed at a single hospital, and hence caution is needed when extending the findings to other demographics, including remote and rural cases. Moreover, this was a retrospective analysis with a limited sample size and a non-randomized study design.

## 5. Conclusions

The present research showed the smartphone-based WeChat platform was highly effective at reducing ischemia time in STEMI patients. This platform can significantly reduce the times for first ECG diagnosis, FMC to CL activity, door to wire, and FMC to wire. Furthermore, the use of the smartphone-based WeChat platform in STEMI patients resulted in a significantly lower rate of heart failure during hospitalization than in the control group. The excellent efficacy and accessibility of WeChat found in this study indicates that social media platforms hold considerable promise for improving the outcomes of cardiovascular disease patients.

## Data Availability

All data points generated or analyzed during this study are included in this article and there are no further underlying data necessary to reproduce the results.
